# Oral Health-Related Quality of Life among Asylum Seekers and Refugees: A Systematic Review

**DOI:** 10.1016/j.identj.2025.103956

**Published:** 2025-10-11

**Authors:** Win Myat Phyo, Duangporn Duangthip, Palinee Detsomboonrat

**Affiliations:** a International Graduate Program in Dental Public Health, Department of Community Dentistry, Faculty of Dentistry, Chulalongkorn University, Bangkok, Thailand; b Division of Dental Public Health, College of Dentistry, The Ohio State University, Columbus, Ohio, USA; c Department of Community Dentistry, Faculty of Dentistry, Chulalongkorn University, Bangkok, Thailand

**Keywords:** Asylum seekers, Refugees, Oral health-related quality of life, Good health and well-being, Reduced inequalities

## Abstract

Asylum seekers and refugees (ASRs) often experience poorer oral health outcomes than the most disadvantaged populations in their host countries. Limited information is available on the oral health-related quality of life (OHRQoL) of ASRs. This systematic review synthesized existing research on OHRQoL among ASRs populations. A systematic search was conducted in EBSCOhost (Dentistry & Oral Sciences Source), PubMed, Scopus, and Google Scholar Advanced Search, without language restrictions. Two researchers independently performed the study selection and data extraction, with any discrepancies resolved through discussion with a third researcher. The citations and references of the included studies were also scrutinized to identify additional relevant literature. The inclusion criteria encompassed experimental or observational studies that evaluated OHRQoL among ASRs using at least one OHRQoL tool. Out of 141 identified, 10 studies met the inclusion criteria. All included studies primarily examined refugee populations, and none focused exclusively on asylum seekers without refugee status. These studies, published between 2014 and 2025, were conducted in United States, Turkey, India, New Zealand, Norway, Jordan, Northern Greece, Austria, and South Australia, with 1844 participants aged 4 to 76 years. Five out of ten studies (50%) measured OHRQoL by using the Oral Health Impact Profile-14. Most of the refugees were from Syria, and Arabic-language OHRQoL tools were the most commonly used (33.3%, 7/21). Physical pain domain emerged as the most frequently impacted domain of OHRQoL, ranging from 28.8% to 70.4%, across the included studies. This review highlights that refugees experience substantial impairments in OHRQoL, with the physical pain domain being the most affected. No studies on OHRQoL focusing exclusively on asylum seekers without refugee status are currently available, highlighting the need for future research to address this evidence gap.

## Introduction

The global scale of forced displacement has reached an unprecedented level. The United Nations High Commissioner for Refugees (UNHCR) indicates that for more than 12 years, the number of individuals forcibly displaced due to persecution, conflict, violence, human rights violations, and significant disruptions to public order has steadily increased, reaching an estimated 122.6 million by the end of June 2024.^1^ Of this total, 43.7 million individuals are classified as refugees, while approximately 8 million are awaiting the outcome of their asylum applications.^1^ As stated by the UNHCR, individuals awaiting a decision on their application for international protection, or who plan to apply for it, are considered asylum seekers. Applying for asylum is a human right, and anyone facing conflict or persecution can seek protection in another country. While not all asylum seekers are ultimately granted refugee status, every recognized refugee has, at some point, sought asylum.^2^

Individuals seeking asylum and those with refugee status often experience prolonged, hazardous, and unstable migration journeys in pursuit of safety. Recognized as a vulnerable population, they often face social marginalization and exclusion.^3^^,^^4^ Compared to the general population, asylum seekers and refugees (ASRs) exhibit more complex health challenges and worse general and emotional health outcomes.^5^ Given their diverse cultural and socioeconomic origins, their understanding, attitudes, and habits regarding oral health can vary widely.^6^ These differences, along with the nature of health care systems and the difficulties faced during migration, can significantly shape their health care-seeking behaviors, perceptions, and access to services.^3^^,^^7^

Determining oral health outcomes predominantly based on the availability and accessibility of oral health care services. For individuals unfamiliar with the structure and protocols of a new health care system, navigating and obtaining oral health care services might provide significant challenges.^8^ Such challenges occasionally elucidate persistent disparities in oral health among vulnerable populations.^9^ ASRs, in particular, face substantial obstacles when trying to obtain oral health care in the countries where they resettle. Keboa et al. (2016) observed the limited access and low utilization of oral health care services among ASRs,^6^ noting that in many host countries, oral health care is often considered an unaffordable luxury.^8^

A systematic review conducted by Paisi et al. (2020) revealed that both demographic factors and health care system characteristics impact how ASRs access oral health care services.^7^ The most common reported barriers include affordability, lack of awareness, and the ability of services to accommodate their needs. These findings underscore the necessity for decision-makers, service planners, and health care professionals to acknowledge and address the unique requirements of these populations. Integrating cultural competence into oral health care provision and related programs is vital to enhancing access.^7^ Supporting the conclusions drawn by Paisi et al. (2020), Wainman et al. (2024) also emphasized 3 primary obstacles faced by ASRs: limited availability of treatments and appointment slots, cultural and linguistic differences, and a lack of oral health knowledge or conflicting beliefs about oral health.^10^

The limited available evidence indicates that the ASRs population generally experiences a higher burden of oral diseases than both the general population and the most socioeconomically disadvantaged groups of the population in their host countries.^6^ Both dental caries and periodontal conditions are prevalent among ASRs,^6^ with a particularly high prevalence of dental caries reported in children.^11^^,^^12^ It is common for ASRs to arrive in their host countries with significant unmet oral health needs, yet their interaction with oral health services is usually reactive, sought mainly in response to acute issues such as severe dental pain.^3^^,^^13^ These circumstances, combined with the broader challenges faced during displacement, render ASRs especially vulnerable to worsening oral health outcomes.^14^ Given that oral health is an integral aspect of general health, poor oral conditions can lead to pain, impaired chewing ability, inadequate dietary intake, and subsequent nutritional deficiencies.^15^ Furthermore, impaired oral health can detrimentally impact oral health-related quality of life (OHRQoL), which is of particular concern for ASRs. Their exclusion from routine health care systems, limited financial resources, poor access to clean water and adequate food, and the breakdown of social support networks all contribute to the adverse effects on their OHRQoL,^16^ which can be measured using various tools designed to capture its multidimensional nature.^17^^,^^18^

Despite the growing attention to ASRs health disparities, OHRQoL remains understudied in these populations due to logistical challenges (e.g., transitory living conditions, language barriers) and a lack of standardized assessment tools adapted to diverse cultural contexts. Existing research predominantly focuses on clinical oral health status rather than quality-of-life impacts, leaving critical gaps in understanding how oral health affects daily functioning and well-being in ASRs. Moreover, to date, no comprehensive systematic review has consolidated the available evidence on OHRQoL among ASRs, which is crucial for public health policymaking and the development of culturally appropriate oral health care interventions. Therefore, this systematic review aims to analyze existing research on OHRQoL among ASRs population.

## Methods

Before initiating the systematic review, a rapid scoping search was carried out to explore existing discussions on the OHRQoL among ASRs and to extract relevant keywords from the available literature. When appropriate, the review protocol was submitted to the International Prospective Register of Systematic Reviews for registration (PROSPERO 2025 CRD420251019305). This systematic review was conducted following the methodological guidance provided in the Joanna Briggs Institute (JBI) Manual for Evidence Synthesis.^19^ The reporting of the review adhered to the guidelines outlined by the Preferred Reporting Items for Systematic Reviews and Meta-Analyses (PRISMA) (Supplementary File 1).^20^

### Eligibility criteria

A comprehensive literature review was conducted utilizing the PICO paradigm, including Participants (P), Intervention (I), Comparison (C), and Outcome (O). This study did not intend to assess the effects of a specific intervention; hence, the “Intervention” and “Comparison” components were not utilized. The review concentrated on delineating the Participants and Outcome. The participants in this study were ASRs at any point in their resettlement process, and the evaluated outcome was their OHRQoL.

All types of original research studies were considered for inclusion. However, studies relying on secondary data such as systematic reviews, research protocols, and similar were excluded. Additionally, book chapters, theses, dissertations, letters, commentaries, editorials, conference presentations, abstracts without full texts, and qualitative studies were not included. Studies including both quantitative and qualitative data, where the quantitative data was pertinent and obtainable, were incorporated. Only those studies that evaluated OHRQoL among ASRs using at least one OHRQoL measurement tool were selected. Studies involving mixed populations, including ASRs and other vulnerable populations like migrants, were included only if the findings specific to ASRs could be separately analyzed. Studies concentrating solely on internally displaced persons (IDPs) were omitted. According to international law, IDPs are distinct from refugees, as they have not crossed international borders. Consequently, their experiences and legal protections vary from those of refugee populations.

### Information sources and search

The following search terms were used on 21 August 2025, across EBSCOhost (Dentistry & Oral Sciences Source), PubMed, and Scopus, to identify articles about asylum seekers, refugees, and oral health-related quality of life: ("Asylum" OR "Asylum Seekers" OR "Refugees") AND ("Oral health-related quality of life" OR "OHRQoL"). The search approach imposed no restrictions on publication date, language, or region to capture differences across various countries and time frames. Furthermore, grey literature was searched via Google Scholar Advanced Search to ascertain possibly pertinent studies. The detailed search strategies used for each database are provided in Supplementary File 2. The citations and references of the included studies were also scrutinized to identify additional relevant literature. Research was gathered through electronic databases, and the literature was organized using EndNote 21, a bibliographic management software.

### Screening process

The screening process was conducted in 2 phases: title/abstract screening and full-text screening. During both phases of the review process, 2 researchers (WMP and PD) conducted independent screenings, and any discrepancies were addressed through discussion or consultation with a third researcher (DD).

### Quality assessment

Two researchers (WMP and PD) independently assessed the quality of the included studies using the Effective Public Health Practice Project (EPHPP) tool for quantitative research.^21^ This tool was created for use within the field of public health and can be utilized in evaluating articles related to any subject within this domain. Any disagreements between them were resolved through consultation with a third researcher (DD). The EPHPP tool evaluates 6 key areas - selection bias, study design, confounders, blinding, data collection methods, and withdrawals and dropouts - by assigning ratings of “strong (1),” “moderate (2),” or “weak (3).” Based on these domains, an overall rating was then determined by the authors using the same 3-point scale.

### Data extraction

Two independent researchers (WMP and PD) carried out data extraction using a pretested data collection form. Any disagreements that arose during the process were addressed by consulting a third researcher (DD), and consensus was reached through discussion.

## Results

The literature review identified 141 studies: 7 from EBSCOhost (Dentistry & Oral Sciences Source), 9 from PubMed, 10 from Scopus, and 115 from Google Scholar Advanced Search. Using EndNote 21, 26 duplicate studies were detected and eliminated, followed by a manual verification. Titles and abstracts of the remaining 115 studies were evaluated based on the inclusion and exclusion criteria, resulting in the deletion of 105 studies that did not align with the study's objectives. Following a comprehensive examination of the full texts of the 10 selected studies, this review excluded one study that did not provide data on OHRQoL for the study population, another study that involved a mixed population of refugees and migrants, as the findings specific to refugees could not be analyzed separately, and a third study that did not use an OHRQoL tool. For the included studies, the citations and references of these studies were also scrutinized to identify additional relevant literature. As a result, 3 additional studies were identified and deemed eligible for inclusion in the review. Therefore, 10 studies were chosen for this systematic review and have been fully identified.

**Fig. 1:**
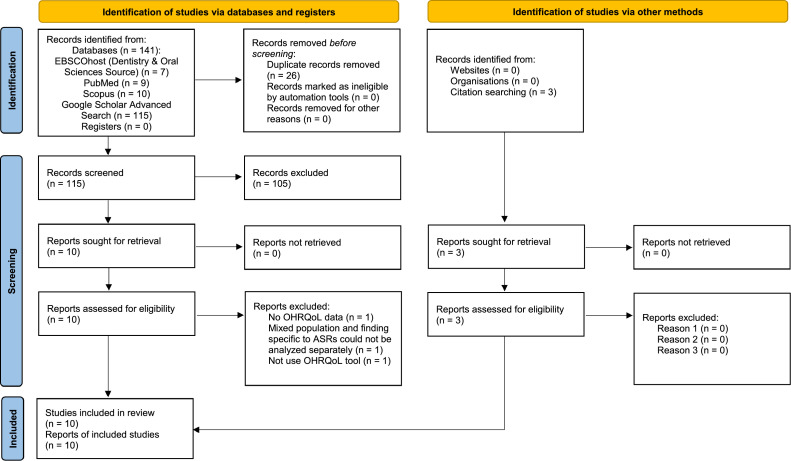
PRISMA 2020 flow diagram for new systematic reviews which included searches of databases, registers and other sources.

Despite the broad search strategy utilized, the final compilation of included studies primarily focused on refugee populations. One study specifically examined individuals with refugee status in Norway, including both resettled refugees and asylum seekers who had been granted permanent residency.^22^ Another study was conducted among individuals from refugee and asylum-seeker backgrounds resettled in South Australia.^23^ No studies focusing exclusively on asylum seekers without refugee status met the inclusion criteria. The studies that were not included during the selection phases were documented, and the selection procedure was outlined (Figure 1) following the 2020 PRISMA flow diagram for systematic reviews.^20^

### Characteristics of the included studies

This systematic review included 10 studies, the key characteristics of which are outlined in Table 1, arranged chronologically by year of publication. Two studies each were published in 2017^16^^,^^24^ and 2022,^14^^,^^25^ while one study was published in each of the following years: 2014,^26^ 2018,^27^ 2019,^22^ 2020,^28^ 2024,^29^ and 2025.^23^ All included studies utilized a cross-sectional design. The ages of 1844 participants spanned from as young as 4 years to as old as 76 years. Regarding sample size, 3 studies involved less than 100 participants,^24^^,^^25^^,^^27^ 3 studies had less than 200,^14^^,^^22^^,^^28^ and the remaining 4 included more than 200 individuals.^16^^,^ ^29^^,^^26^^,^^23^ The overall sample sizes ranged between 33 and 471. The studies were conducted in United States,^26^ Turkey,^24^ India,^16^^,^^25^ New Zealand,^27^ Norway,^22^ Jordan,^28^ Northern Greece,^14^ Austria,^29^ and South Australia.^23^ In terms of participant eligibility based on length of stay, 4 studies did not provide specific criteria.^14^^,^^24^^,^^27^^,^^25^ One required participants to have stayed for a minimum of 4 months,^16^ another set a minimum of 12 months,^28^ and 4 specified a maximum duration of 2 years,^22^ 7 years^23^ and 10 years.^26^^,^^29^

**Table 1:** Characteristics of participants in the included studies.

Author (Year)	Study design	Sample size	Age in year (range/ mean ± SD)	Participants' origin	Duration of stay	Study location and country	OHRQoL tool	Language
Geltman et al. (2014)	Cross-sectional	439	(35.2 ± 14.7)	Somalia, Kenya, Ethiopia, and others	Not more than 10 years	(Boston, Lynn, Chelsea, Worcester and Springfield) Massachusetts, United States	New Brief Measure OQOL	Somali
Pani et al. (2017)	Cross-sectional	42	(38.2 ± 8.1)	Syria	NR	Kirikhan, Turkey	P-CPQ-8	Arabic
Niraj et al. (2017)	Cross-sectional	201	4 to 76 (21.36 ± 16.30)	Myanmar	Over 4 months in New Delhi, India	Refugee Camps, KalindiKunj (ShaheenBagh, JJ Colony, BodolaGoung and Hastall), New Delhi, India	OHIP-14	Hindi
Barazanchi et al. (2018)	Cross-sectional	62	24 to 60 (37.7)	Syria	NR	Community English class in Dunedin, New Zealand	OHIP-14	Arabic
Høyvik et al. (2019)	Cross-sectional	132	(31.8 ± 11.6)	Middle East (Syria, Iran, Iraq, Afghanistan) and Africa (Eritrea, Somalia, Sudan, Nigeria)	Not more than 2 years in Norway	Regular centers within commuting distance to Oslo, Norway	OIDP	Arabic, Sorani, Tigrinya, Amharic, Somali, Norwegian, or English
Abu-Awwad et al. (2020)	Cross-sectional	102	16 to 64 (34 ± 10)	Syria	At least 12 months in the camp in Jordan (1.75 ± 0.89) years Range: 1.1- 3.8 years	Azraq refugee camp, Jordan	OHRQoL-UK	Arabic
Zaheer et al. (2022)	Cross-sectional	156	18 to 64	Iraq and others	No minimum period (<1, 1 - 6, >6) months	Northern Greece (Kilkis, Derveni (Alexil), Alexandreia, Loutra Volvis, Nea Kavala, Softex and Sinatex)	ADAHPI (Not validated)	English, Arabic, or Farsi
Murali et al. (2022)	Cross-sectional	33	≥ 18	Myanmar	NR	Refugee camp Kelambakkam, Chennai, India	OHIP-14	NR
Fink et al. (2024)	Cross-sectional	471	15 to 75 (Median (IQR): 23 (20-29)	North Africa (Libya, Egypt, Morocco, Tunisia, Algeria, Sudan), East Africa (South Sudan, Tanzania), Arabian Peninsula (Yemen, Qatar, Oman, Saudi Arabia, United Arab Emirates, Kuwait), Middle East (Iraq, Jordan, Lebanon, Syria, Israel), and others	Maximum of 10 years in Austria	Austria	OHIP-14	German and Arabic
Ziersch et al. (2025)	Cross-sectional	206	18 to 73 (35.49)	Syria, Iran, Afghanistan, Iraq, Pakistan	< 7 years in Australia	South Australia	OHIP-14	English, Arabic, Farsi, or Dari

*ADAHPI*, American Dental Association Health Policy Institute oral health and well-being questionnaire (9 items); *IQR*, inter quartile range; *New Brief Measure OQOL*, new brief measure oral quality of life; *NR*, not reported; *OHIP-14*, oral health impact profile (14 items); *OHRQoL-UK*: United Kingdom Oral Health Related Quality of Life measure (16 items); *OIDP*, oral impact on daily performance (8 items); *P-CPQ-8*, parental-caregiver perceptions questionnaire-8 (8 items).

Five of the ten studies concentrated on refugee groups of a single nationality - Syrian from Syria^24^^,^^27^^,^^28^ or Rohingya from Myanmar.^16^^,^^25^ Another study was conducted among Somali refugees living in Massachusetts, United States who came from Somalia, Kenya, Ethiopia, and other countries.^26^ A study conducted in Norway focused on refugees originating from the Middle East, particularly Syria, Iran, Iraq, and Afghanistan, as well as from African countries including Eritrea, Somalia, Sudan, and Nigeria. The majority of participants were from Syria and Eritrea.^22^ One study conducted in Northern Greece mentioned Iraqi-Kurdish refugees in Loutra Volvis, but did not identify the refugee groups in the other study locations.^14^ Another study encompassed refugees from a variety of regions, such as the Middle East (Iraq, Jordan, Lebanon, Syria, and Israel), East Africa (South Sudan and Tanzania), North Africa (Libya, Egypt, Morocco, Tunisia, Algeria, and Sudan), the Arabian Peninsula (Yemen, Qatar, Oman, Saudi Arabia, United Arab Emirates, and Kuwait), and other areas, with 77.1% being from Syria.^29^ In addition, a study conducted in South Australia included refugees from Syria, Afghanistan, Iran, Iraq, and Pakistan, most of whom were from Syria and Iran.^23^

Almost all included studies utilized validated tools to assess OHRQoL^26^^,^^24^^,^^16^^,^^27^^,^^22^^,^^23^^,^^25^^,^^28^^,^^29^ with only one study employing a non-validated tool.^14^ Among these, 5 studies used the Oral Health Impact Profile-14 (OHIP-14) tool to assess OHRQoL.^16^^,^^23^^,^^25^^,^^27^^,^^29^ Each of the New Brief Measure of Oral Quality of Life (OQOL),^26^ the short form of Parental-Caregiver Perceptions Questionnaire-8 (P-CPQ-8),^24^ the Oral Impacts on Daily Performance (OIDP),^22^ the United Kingdom Oral Health-Related Quality of Life (OHRQoL-UK),^28^ and the Oral Health and Well-Being questionnaire from the American Dental Association Health Policy Institute (ADAHPI)^14^ tools was utilized in a single study. Five studies applied OHRQoL assessments in a single language (Somali,^26^ Arabic,^24^^,^^27^^,^^28^ or Hindi^16^), one used 2 languages (German and Arabic),^29^ one used 3 languages (English, Arabic, and Farsi),^14^ one used 4 languages (English, Arabic, Farsi, or Dari),^23^ and another used multiple languages (Arabic, Sorani, Tigrinya, Amharic, Somali, Norwegian, and English).^22^ Only one study did not specify the language used in the OHRQoL assessment.^25^ Arabic was the most translated language among the OHRQoL tools used, comprising 33.3% (7/21) of all language instances across the studies. Some studies reported that the tools had been validated in the languages used.^24^^,^^27^, ^28^, ^29^

### OHRQoL among refugees

Several studies utilizing a range of OHRQoL assessment tools have revealed persistent oral health challenges faced by refugees (Table 2). The findings are organized by the specific tools employed in the evaluations.

**Table 2:** Negatively impacted oral health-related quality of life among participants in the included studies.

Author (Year)	OHRQoL Tool	Standard Score Range	Overall Mean (SD)	Percentage (n) of participants negatively impacted on their OHRQoL														
Geltman et al. (2014)	New Brief Measure OQOL	NR	1.2 (0.3)	NR														

Pani et al. (2017)	P-CPQ-8		Father: 14.50 (5.88) Mother: 18.19 (6.28)	**Oral Symptoms**			**Emotional well-being**				**Functional Limitations**				**Social well-being**			
				**Pain in the teeth, lips, jaws or mouth**	**Food caught in or between the teeth**		**Been upset**		**Been irritable or frustrated**		**Difficulty biting or chewing firm foods**		**Taken longer than others to eat a meal**		**Missed school or preschool**		**Not wanted to talk to other children**	

				58.9% (25)	36% (15)		54% (22)		34.3% (14)		38.7% (16)		30.9% (13)		48.1% (19)		41.4% (17)	

				**Functional Limitations**		**Physical Pain**			**Psychological Discomfort**		**Physical Disability**		**Pshychological Disability**		**Social Disability**		**Handicap**	
Barazanchi et al. (2018)	OHIP-14	0-56	19.2 (14.3)	21.70%		70.40%			47.30%		36.50%		39.70%		21.20%		24.10%	

Ziersch et al. (2025)	OHIP-14	0-56	20.9 (14.1)	9.2% (38)		53.3% (105)			44.9% (89)		34.7% (68)		29.9% (59)		34.2% (67)		28.1% (55)	

				**Had difficulties pronouncing words**	**Felt Limitation in sense of taste**	**Had pain in oral region**		**Felt uncomfortable eating certain foods**	**Felt insecure related to teeth/denture**	**Felt tense because of problem**	**Felt diet has been unsatisfactory**	**Had to interrupt meals**	**Felt difficulties relaxing**	**Felt embarrassed**	**Felt irritated by others**	**Had difficulty with daily routine**	**Felt life in general was less satisfying**	**Been totally unable to function**

Niraj et al. (2017)	OHIP-14	0-56	19.18 (2.19)	94.6% (106)	100% (112)	97.4% (109)		100% (112)	94.6% (106)	100% (112)	100% (112)	100% (112)	100% (112)	100% (112)	100% (112)	100% (112)	100% (112)	100% (112)

Murali et al. (2022)	OHIP-14	0-70	67.47 (2.26)	6.1% (2)	12.1% (4)	27.3% (9)		9.1% (3)	6.1% (2)	6.1% (2)	9.1% (3)	6.1% (2)	6.1% (2)	6.1% (2)	6.1% (2)	9.1% (3)	6.1% (2)	6.1% (2)

Fink et al. (2024)	OHIP-14	0-56	Median (IQR) 8 (2-17)	21.40%	15.60%	65.50%		46.70%	53.90%	38.60%	41.40%	40.40%	43.50%	41.60%	36.90%	33.60%	38.20%	21.70%

Høyvik et al. (2019)	OIDP	(8)-40		**Problems in eating and enjoying food**	**Problem in speaking and pronouncing clearly**		**Problem in tooth cleaning**		**Problems in sleep and relaxation**		**Problems in smiling and showing teeth without being embarrassed**		**Problmes in being emotionally stable**		**Problems in being sociable (enjoying being with other people)**		**Problems in performing daily work/daily chores**	

			Middle East: 30.4 (10)	44.40%	15.50%		35.50%		24.40%		28.90%		20.00%		22.20%		17.70%	

			Africa: 33.3 (7.4)	34.40%	8.00%		29.90%		9.10%		17.20%		9.10%		8.00%		8.00%	

Abu-Awwad et al. (2020)	OHRQoL-UK	16-80	56.6 (7.8)	**Physical domain**					**Social domain**					**Psychological domain**				

				28.80%					25.40%					24.40%				

Zaheer et al. (2022)	ADAPHI	NR	NR	**Difficulty chewing**	**Difficulty with speech**		**Dry mouth**	**Felt anxious**	**Felt embarrassment**		**Avoided smiling**		**Difficulty doing daily activity**		**Difficulty sleeping**		**Experienced pain**	

				54.3% (80)	23.65% (35)		50.71% (71)	34.93% (51)	64.63% (95)		48.98% (72)		33.10% (48)		47.95% (70)		71.5% (105)	

NR, not reported.

#### New brief measure of OQOL

This new brief measure of OQOL tool was developed by adapting existing OHRQoL tools, including the Geriatric Oral Health Assessment Instrument (GOHAI), Oral Health Quality of Life (OHQOL), and OHIP, to serve as a public health tool for evaluating the effects of oral health conditions on individuals and populations.^30^ This OQOL tool is available in 2 versions: a 5-domain version (covering impairment, physical, distress, worry, and role) with a 5-item Oral Quality of Life Scale and a Denture Scale, as well as a 12-item Short Form Oral Quality of Life Measure.^30^ In a study of Somali refugees living in Massachusetts, United States, Geltman et al. (2014) reported an overall mean (SD) of 1.2 (0.3). However, the study did not specify the results for individual domains or items, nor did it indicate which version of the New Brief Measure of OQOL had been applied.^26^

#### P-CPQ-8

The P-CPQ-8 tool encompasses 4 distinct domains designed to evaluate different aspects of OHRQoL, namely oral symptoms, emotional well-being, functional limitations, and social well-being. In the study by Pani et al.,^24^ the overall mean scores for the P-CPQ-8 were reported as 14.50 (SD = 5.88) for fathers and 18.19 (SD = 6.28) for mothers, without a significant gender difference. The investigation, encompassing 4 domains, highlighted that the oral symptoms domain had the highest mean score at 4.56 (± 2.2), signaling significant concerns regarding pain, with only 38.1% of participants claiming they never faced discomfort in their teeth, lips, jaws, or mouth. In contrast, the functional limitations domain exhibited the lowest mean score, as more than 60% of respondents stated they never experienced difficulties in biting or chewing firm foods (61.9%) or in taking longer than others to complete a meal (64.3%).

#### OHIP-14

The OHIP-14 short form evaluates multiple aspects of OHRQoL across 7 categories. These domains include functional limitations, physical pain, psychological distress, physical disability, psychological disability, social disability, and handicap, offering a thorough assessment of the influence of oral health on individuals' daily lives and overall well-being. Among the studies, only 2 reproted the prevalence of OHIP-14 at the domain level,^23^^,^^27^ while the remaining 3 presented it at the item level.^16^^,^^25^^,^^29^

A study by Barazanchi et al.^27^ reported a comparable overall mean score of 19.2 (SD = 14.3) and 78% of participants often or very often experienced at least one impacts, with a high prevalence of physical pain domain observed in 70.4% of the participants. Additionally, social disability was found to have the least impact, affecting only 21.2% of participants. It is important to note that not all participants completed every question; specifically, only 41 out of the 62 participants answered all items. Nonetheless, the analysis was conducted based on the available responses, indicating that the data for each question may represent different numbers of participants.

In the study by Ziersch et al.,^23^ the overall mean (SD) was reported as 20.90 (14.10). The domain most impacted was physical pain (53.3%), succeeded by psychological discomfort (44.9%), physical disability (34.7%), social disability (34.2%), psychological disability (29.9%), and handicap (28.1%), while functional limitation was the least affected (9.2%).

Niraj et al.^16^ reported an overall mean (SD) OHRQoL score of 19.18 (5.282). The psychological disability dimension demonstrated the highest mean score of 3.23 (0.455), indicating significant psychological impacts. Conversely, the physical disability domain exhibited the lowest mean score, measured at 2.34 (0.566). Significantly, 97.4% of participants indicated suffering painful aching in their oral cavities. Moreover, all participants reported limitation in taste perception, discomfort while eating, and feeling of tension because of problem. Moreover, all participants recognized difficulties across all items within the domains of physical disability, psychological disability, social disability, and handicap. In this study, only 112 of the 201 participants responded to all items of the OHIP-14.

A study by Murali et al.^25^ reported that overall mean (SD) of 67.47 (2.26), with the highest prevalence of pain in oral region (27.3%), then followed by the felt limitation in sense of taste (12.1%). A prevalence of 9.1% was noted for difficulties such as feeling uncomfortable when eating certain foods, perceiving the diet as unsatisfactory, and experiencing disruptions in daily routine. Additionally, 6.1% of respondents reported challenges including difficulty in word pronunciation, interruptions during meals, feelings of irritation with others, and across all items within the domains of psychological discomfort, psychological disability, and handicap. However, the limited sample of 33 participants in this study could affect the reliability of the OHRQoL findings.

In the study by Fink et al.,^29^ the median OHRQoL score was reported to be 8, with an interquartile range (IQR) of 2 to 17. The analysis indicated that physical pain was the domain with the greatest impact, resulting in a median score of 2 (IQR: 0-4). Notably, only 34.4% of participants reported no pain in the oral region, and just 53.4% indicated that they did not experience discomfort when consuming certain foods. In contrast, the functional limitation domain exhibited the least degree of impairment, with a median score of 0 (IQR: 0-1); only 21.4% of participants experienced difficulties in pronouncing words, and 15.6% reported a limitation in their sense of taste. Notably, only 401 of the 471 participants completed all items of the OHIP-14.

Studies utilizing the OHIP-14 consistently identified physical pain as a major concern among refugees. Both Barazanchi et al.^27^ and Ziersch et al.^23^ emphasized its significance, and the remaining 3 studies corroborated these findings by also reporting oral pain as the most frequently affected issue.^16^^,^^25^^,^^29^ Psychological factors were also notably impacted, evident from elevated scores in the psychological discomfort and psychological disability domains, which reflect the mental health challenges encountered by this group.

#### OIDP

A study by Høyvik et al.^22^ utilized the OIDP tool, comprising 8 questions concerning the frequency of challenges associated with dental concerns or denture complications encountered in the past 6 months while executing ordinary daily activities. The results revealed that 50.4% of participants experienced at least one weekly OIDP, with a sum score of 30.4 (SD 10.0) for Middle Eastern participants and 33.3 (SD 7.4) for those from African countries. A substantial proportion of participants reported experiencing problems with their teeth or dentures affecting their ability to eat and enjoy food (37.9%) and difficulties with tooth cleaning (31.8%) on a weekly basis or more frequently. Participants from the Middle East demonstrated statistically significant higher dissatisfaction with the conditions of their teeth and reported more substantial difficulties concerning sleep, relaxation, and social interaction due to dental issues compared to their African counterparts.

#### OHRQoL-UK

Abu-Awwad et al.^28^ employed the OHRQoL-UK tool which comprises 3 key domains: physical, social, and psychological and documented a mean total score of 56.6 (SD = 7.8), with scores ranging from 32 to 80. Their domain-specific evaluation indicated considerable adverse effects, with mean scores of 3.45/5 for the physical domain, 3.5/5 for the social domain, and 3.67/5 for the psychological domain. Among the participants, 28.8% experienced negative impact on physical domains, 25.4% encountered difficulties in social domain, and 24.4% reported issues related to psychological domain, indicating a slightly greater negative influence on physical aspects compared to psychological ones. Additionally, a substantial proportion of participants experienced negative impacts on daily activities, including challenges with eating or enjoying food (52%), discomfort or pain (48%), and reduced ability to maintain work performance or carry out routine responsibilities (39.2%).

#### ADAHPI

In the study by Zaheer et al.^14^ utilizing the ADAHPI questionnaire, which incorporates OHRQoL items across several domains including challenges with chewing, speech difficulties, xerostomia, anxiety, embarrassment, smiling avoidance, limitations in daily activities, sleep disturbances, and pain, with the most frequently reported concerns were pain (71.5%), difficulty in chewing (54.3%), and dry mouth (50.7%). These results highlight the substantial burden these oral health issues impose on individuals’ daily activities and their overall quality of life.

### Quality assessment

The methodological quality across all studies was deemed weak due to 2 or more weak ratings among the 6 assessed components, resulting in an overall weak classification (Table 3). Selection bias was primarily rated as moderate, suggesting that while participants were somewhat representative of the target population, most studies reported a strong willingness to participate. The quality of study design and blinding was regarded as weak, since all studies utilized a cross-sectional design, and participants were aware of the research questions being investigated. Additionally, considering confounding variables was rated mainly as weak, with most studies failing to conduct regression analyses to adjust for these factors. In terms of data collection methods, nearly all studies were strong, except one that employed a non-validated tool, and another for which back-translation of the OIDP inventory was not feasible.

**Table 3:** Summary of quality assessment of included studies using EPHPP tool.

Author/Year	Selection bias	Study design	Confounders	Blinding	Data collection method	Withdrawals and dropouts	Global rating
Geltman et al. (2014)	2	3	3	3	1	Not applicable	3
Pani et al. (2017)	2	3	3	3	1	Not applicable	3
Niraj et al. (2017)	2	3	3	3	1	Not applicable	3
Barazanchi et al. (2018)	2	3	3	3	1	Not applicable	3
Høyvik et al. (2019)	2	3	2	3	2	Not applicable	3
Abu-Awwad et al. (2020)	2	3	2	3	1	Not applicable	3
Zaheer et al. (2022)	2	3	3	3	3	Not applicable	3
Murali et al. (2022)	2	3	3	3	1	Not applicable	3
Fink et al. (2024)	2	3	2	3	1	Not applicable	3
Ziersch et al. (2025)	2	3	2	3	1	Not applicable	3

EPHPP, effective public health practice project.

**EPHPP rating**: Strong (1), Moderate (2), Weak (3).

## Discussion

To the best of our knowledge, this systematic review appears to be the first of its kind to comprehensively examine OHRQoL within the population of ASRs. By synthesizing existing evidence, this review fills a critical gap in the literature and provides a foundation for future research and interventions. The accumulation of evidence from this review underscores the significant oral health challenges that refugees encounter, characterized by increased levels of pain and psychological distress. The results emphasize an urgent need for targeted interventions aimed at enhancing access to oral health care and addressing the specific requirements of this vulnerable population.

In this review, a consistent pattern of poor OHRQoL emerged, with the physical pain domain identified as the most commonly affected aspect among refugees. The prevalence of physical pain domain within the OHRQoL domains ranged from 28.8% to 70.4%, while reports of pain in the oral cavity more broadly ranged from 27.3% to 97.4% across the included studies. These findings highlight the significant burden of oral health problems in these populations, aligning with Kateeb and Lee,^31^ who argue that oral health is a basic human right for refugees, frequently compromised by systemic obstacles like restricted dental care access, economic limitations, and cultural or linguistic barriers. They highlight that refugees frequently face dental caries and periodontal diseases, which lead to physical discomfort and reduced OHRQoL. Consistent with our review, physical pain was a recurring concern across studies, highlighting the urgent need for affordable and accessible dental care.

This review also highlighted several critical factors that have a significant influence on OHRQoL among refugees. Across the studies, the negative effects on OHRQoL are closely linked to the location and duration of settlement, the individuals’ country of origin, smoking habits, the frequency of toothbrushing, usage of fluoride toothpaste, self-perceived oral health, the recency of dental visits, and financial satisfication.^14^^,^^22^^,^^23^^,^^28^^,^^29^ Specifically, brushing teeth twice daily was correlated with enhanced OHRQoL.^29^ Notably, new arrivals in the settlements reported a greater adverse effect on their OHRQoL.^14^ It was observed that older refugees are particularly susceptible to the negative implications of poor oral health on their quality of life.^28^ Furthermore, dental pain could serve as an indicator of potential psychological issues.^24^ Despite these significant observations, the studies utilized a range of statistical methods to draw their conclusions, which made the overall findings overarching. They also possess inherent limitations, such as challenges in establishing associations and variations in data interpretation. The different methods used can affect how similar the results are between studies, highlighting the need for consistent research methods to better meet the oral health needs of refugees.

Oral health issues are becoming more and more recognized for their many impacts on individuals' overall quality of life and well-being. Although they do not replace the evaluation of diseases or treatment outcomes, quality of life measures are rather important in supporting role.^32^ However, the complexity and subjectivity of OHRQoL, shaped by individual perceptions and experiences, complicate its assessment. Furthermore, none of the existing evaluation measures encompass the entire range of characteristics associated with OHRQoL.^32^ Furthermore, while evaluating the OHRQoL of refugees, it is essential to take into account the stressors associated with displacement and the adaptation to unfamiliar cultural contexts.

Five out of ten studies (50%) in this review employed the OHIP-14, a well-validated tool encompassing 7 categories, to assess OHRQoL.^16^^,^^23^^,^^25^^,^^27^^,^^29^ All studies employed a 5-point Likert scale to capture participant responses; however, Murali et al (2022) included an additional ‘don’t know’ option. Minor discrepancies were observed acorss studies in the operationalization of response categories regarding the description and labelling of scale points. Moreover, there were variations in the administration of the OHRQoL instruments: 4 studies employed interviews for data collection,^16^^,^^22^^,^^25^^,^^26^ whereas the rest utilized self- reported measures.^14^^,^^23^^,^^24^^,^^27^, ^28^, ^29^ An interview-based measure was used to minimize incomplete responses and data loss potentially associated with participants' educational backgrounds,^16^^,^^25^ while also promoting inclusivity and preventing any bias or stigmatization toward those who are illiterate.^22^ In some of the included studies, interpreters or translators were provided either to assist illiterate participants or to facilitate communication with participants who spoke different languages.^14^^,^^22^^,^^27^ A study employed an online survey style as one of the self-reported methodologies.^29^ Significantly, the other studies gathered data directly from participants,^14^^,^^16^^,^^22^^,^^23^^,^^25^, ^26^, ^27^, ^28^, ^29^ but only one study assessed OHRQoL from parental perceptions of their children.^24^

Moreover, considering the original geographical background of the included participants, several included studies used translated versions of OHRQoL tools; Arabic being the most translated language used in these regard.^14^^,^^22^, ^23^, ^24^^,^^27^, ^28^, ^29^ This emphasizes the need of cross-cultural adaptation in guaranteeing that OHRQoL tools are conceptually and linguistically consistent across diverse cultures. Interpreting OHRQoL results across different contexts necessitates meticulous methodological considerations, as demonstrated by the observed discrepancies in administration modes and the utilization of modified tools. The living arrangements for refugees vary based on whether the host countries or international agencies support them; they may reside in dedicated camps, shelters, or within local communities. Generally, those resettled in industrialized countries tend to receive more comprehensive services, including oral health care, compared to refugees residing in developing countries.^6^ Therefore, in this review, refugees from United States, New Zealand, Norway, Austria, Northern Greece, and South Australia may face fewer barriers to oral health care and have better OHRQoL than those in developing countries such as Turkey, India, and Jordan. This review exclusively examined the OHRQoL of refugees, so the findings may not be applicable to migrants or other vulnerable demographic groups.

Refugees from diverse backgrounds often experience inadequate oral health, significantly affecting their OHRQoL. Those who have recently arrived may lack awareness about the link between oral and general health, as well as the value of routine dental visits, even in the absence of pain or urgent issues. This review identifies critical evidence to inform policy development aimed at promoting oral health equity among ASRs.

The findings of this systematic review highlight an urgent need for multilevel, culturally adapted interventions to address the significant oral health disparities among ASRs. Oral health assessments should be integrated into mandatory initial medical screenings for ASRs, prioritizing pain management and urgent care, given the high prevalence of untreated dental pain. Health care providers require training to recognize oral health disparities (e.g., caries, periodontal disease) and their systemic links (e.g., diabetes, malnutrition), as poor oral health exacerbates existing vulnerabilities in this population. Also, access to culturally competent care must be expanded through mobile dental clinics or partnerships with community health centers in high-density ASRs areas, complemented by professional interpreters and translated educational materials (for instance, Arabic, Rohingya) to overcome language barriers, a critical step given that participants in 7 of the 10 included studies spoke Arabic.

Moreover, preventive programs should collaborate with Non-Governmental Organizations (NGOs) to distribute oral hygiene kits in camps and resettlement communities, coupled with peer-led workshops that address cultural beliefs while teaching evidence-based practices. Such interventions are vital for new arrivals, who reported worse OHRQoL in this review. Additionally, advocacy efforts must urge policymakers to include oral health in refugee health strategies, prioritizing vulnerable groups like pregnant women, children, and asylum seekers who face exclusion due to uncertain legal status, and ensure equitable access to essential oral health services as a right through partnerships with national and local health organizations.^31^^,^^33^ National refugee health databases should track oral health outcomes for equitable resource allocation. These measures, supported by longitudinal research on asylum seekers, a critically understudied subgroup, would align with the World Health Organization’s 2030 oral health goals and help mitigate the systemic inequities identified in this review.

One of the main strengths of this review is the participation of researchers at every phase of the screening and reviewing process, reducing selection bias. The study selection was conducted systematically and comprehensively, reducing the risk of omitting relevant evidence. However, 3 studies were omitted. One assessed a preventative outreach educational intervention to improve dental caries outcomes and OHRQoL among refugee children.^34^ However, it lacked OHRQoL data expressed as percentage of negatively impacted OHRQoL, means/medians and standard deviations, which excluded it. Another study involved a mixed group of migrant and refugee children, but it was not included since data particular to refugees could not be separated.^35^ The third, conductued among Rohingya refugees in Cox’s Bazar, Bangladesh, was also excluded as it did not empoly an OHRQoL tool, although it reported a high prevalence of dental pain (77.8%), functional difficulties (85.3%), and problems sleeping (51.6%).^36^ These exclusions increase the likelihood of excluding certain relevant data, supporting this review's results more or less exclusively.

This review has several limitations that warrant consideration. The methodological quality of included studies, as assessed by the EPHPP tool, was uniformly weak due to cross-sectional designs, lack of blinding, and inadequate adjustment for confounders (e.g., socioeconomic status, trauma history). These factors limit causal inferences and may introduce bias, particularly in self-reported outcomes. Missing data in some studies (e.g., Barazanchi et al., 2018, where only 41 of 62 participants completed all OHIP-14 items) could skew prevalence estimates of OHRQoL impacts.

Despite our inclusive search strategy, the review predominantly captured data on refugees with permanent status, revealing a critical evidence gap for asylum seekers, a population likely facing even greater barriers due to uncertain legal status and restricted health care access. This imbalance underscores the need for targeted research on asylum seekers, who are systematically underrepresented in oral health literature. The heterogeneity in OHRQoL tools (e.g., OHIP-14 vs. OIDP) and administration methods (interviews vs. self-report) precluded meta-analysis and complicated cross-study comparisons. Future studies should employ longitudinal designs, standardized tools, and stratified analyses by legal status (refugee vs. asylum seeker) to clarify disparities.

Notably, this systematic review aligns with the core principle of the United Nations' Sustainable Development Goals (SDGs): "leave no one behind” by addressing a critical gap in global health equity through its focus on OHRQoL among ASRs. Highlighting the oral health challenges among these populations, often overlooked by conventional health care systems, supports the objectives of SDG 3 (Good Health and Well-Being) and SDG 10 (Reduced Inequalities) by raising awareness and informing targeted efforts to address these goals. Establishing health policies and programs that are inclusive and cater to the needs of vulnerable populations necessitates the integration of this knowledge.

## Conclusion

In conclusion, the evidence presented in this review indicates that individuals with refugee backgrounds experience a lower OHRQoL. This study represents the inaugural systematic review of OHRQoL among ASRs, underscoring the critical necessity to address oral health disparities within this vulnerable group. Our findings establish a fundamental basis for future research, policy, and practice focused on enhancing overall well-being and urge host countries to address these issues.

## Authors contribution

**Win Myat Phyo**: Conceptualization, Methodology, Data Analysis and Interpretation, Writing – original draft. **Duangporn Duangthip**: Conceptualization, Methodology, Analysis and Interpretation, Writing – review and editing. **Palinee Detsomboonrat**: Conceptualization, Methodology, Data Analysis and Interpretation, Writing – review and editing. All authors gave their final approval and agreed to be accountable for all aspects of the work.

## Conflict of interest

The authors declare that they have no known competing financial interests or personal relationships that could have appeared to influence the work reported in this paper.

## Appendix Supplementary materials

Image, application 1

Image, application 2

## Footnotes

Supplementary material associated with this article can be found in the online version at doi:10.1016/j.identj.2025.103956.
