# Supplementary material for: Oral Health-Related Quality of Life among Asylum Seekers and Refugees: A Systematic Review
Source: Int Dent J. 2025 Oct 11;75(6):103956. doi: 10.1016/j.identj.2025.103956 (PMC12547705; doi:10.1016/j.identj.2025.103956)
Supplement: Supplementary file 2 [file mmc2.docx]

**Supplementary File 2: Search Strategies used for each database**

| **Database** | **Search strategies** |
| --- | --- |
| EBSCOhost (Dentistry & Oral Sciences Source) | (asylum or asylum seeker or refugee) (All fields)  AND  (oral health-related quality of life or OHRQoL) (All fields) |
| Scopus | ( ( TITLE-ABS-KEY ( "asylum" OR "asylum seeker" OR "refugee" ) ) AND TITLE-ABS-KEY ( ( "oral health-related quality of life" OR "OHRQoL" ) ) ) |
| PubMed | ("asylum"[All Fields] OR "asylum seeker"[All Fields] OR "refugee"[All Fields]) AND ("oral health-related quality of life"[All Fields] OR "OHRQoL"[All Fields]) |
| Google Scholar Advanced Search | With all of the words- OHRQoL  With the exact phrase- oral health related quality of life  With at least one of the words- asylum “asylum seeker” refugee  Where my words occur- anywhere in the article |
